# The Zinc Transporter Zip7 Is Downregulated in Skeletal Muscle of Insulin-Resistant Cells and in Mice Fed a High-Fat Diet

**DOI:** 10.3390/cells8070663

**Published:** 2019-07-01

**Authors:** Shaghayegh Norouzi, John Adulcikas, Darren C Henstridge, Sabrina Sonda, Sukhwinder Singh Sohal, Stephen Myers

**Affiliations:** 1 College of Health and Medicine, School of Health Sciences, University of Tasmania, Hobart, Tasmania 7005, Australia; 2 Baker Heart and Diabetes Institute, Melbourne, Victoria 3004, Australia

**Keywords:** zinc, zinc transporter, Zip7, insulin signaling pathway, high-fat diet, skeletal muscle

## Abstract

Background: The zinc transporter Zip7 modulates zinc flux and controls cell signaling molecules associated with glucose metabolism in skeletal muscle. The present study evaluated the role of Zip7 in cell signaling pathways involved in insulin-resistant skeletal muscle and mice fed a high-fat diet. Methods: Insulin-resistant skeletal muscle cells were prepared by treatment with an inhibitor of the insulin receptor, HNMPA-(AM)3 or palmitate, and Zip7 was analyzed along with pAkt, pTyrosine and Glut4. Similarly, mice fed normal chow (NC) or a high-fat diet (HFD) were also analyzed for protein expression of Glut4 and Zip7. An overexpression system for Zip7 was utilized to determine the action of this zinc transporter on several genes implicated in insulin signaling and glucose control. Results: We identified that Zip7 is upregulated by glucose in normal skeletal muscle cells and downregulated in insulin-resistant skeletal muscle. We also observed (as expected) a decrease in pAkt and Glut4 in the insulin-resistant skeletal muscle cells. The overexpression of Zip7 in skeletal muscle cells led to the modulation of key genes involved in the insulin signaling axis and glucose metabolism including *Akt3*, *Dok2*, *Fos*, *Hras*, *Kras*, *Nos2*, *Pck2*, and *Pparg*. In an *in vivo* mouse model, we identified a reduction in Glut4 and Zip7 in the skeletal muscle of mice fed a HFD compared to NC controls. Conclusions: These data suggest that Zip7 plays a role in skeletal muscle insulin signaling and is downregulated in an insulin-resistant, and HFD state. Understanding the molecular mechanisms of Zip7 action will provide novel opportunities to target this transporter therapeutically for the treatment of insulin resistance and type 2 diabetes.

## 1. Introduction

It was estimated in 2013 that 382 million people were affected by diabetes and this number is expected to increase to 592 million by 2035 [1]. This rapid escalation in diabetes can be attributed to rapid economic developments and lifestyle changes associated with reduced physical activity and an increase in the consumption of high calorie diets, resulting in a higher obesity prevalence [2]. Adipose tissue dysfunction is one mechanism responsible for systemic metabolic complications, such as type-2 diabetes (T2D) [3,4]. T2D is characterized by insulin resistance in major peripheral metabolic tissues including adipose tissue, liver and skeletal muscle. While most obese individuals do not become diabetic even in the face of a high degree of insulin resistance, most patients with T2D are obese [5]. Thus, in addition to insulin resistance, compromised pancreatic β-cell function leads to increased blood glucose and contributes to hyperglycemia and overt T2D [6].

Research on T2D has revealed a role for the physiological importance of zinc and the proteins that transport this metal ion in cells (zinc transporters) in diseases associated with abnormal cell signaling pathways and metabolism such as insulin resistance (IR) and T2D [7]. Zinc is a vital trace element that is present in organs, tissues, fluids and secretions and functions as a critical cofactor in an extensive number of biological signaling pathways as a catalytic, structural or regulatory component [8]. The storage, release and distribution of cellular zinc are regulated by a family of zinc transporter proteins and metallothioneins. In mammals, there are 24 zinc transporter proteins divided into two families. These are the zinc efflux (Slc30/ZnT, members 1–10) and the zinc influx (Slc39/ZIP, members 1–14) proteins [9]. The ZnT members function to transport zinc from the cell or into subcellular organelles when cytoplasmic zinc is high. In contrast, the ZIP members function to transport zinc into the cell when zinc in the cytosol is low or depleted [10].

Emerging research has highlighted key roles for zinc transporter ZIP7 in signaling pathways. ZIP7 is located in the endoplasmic reticulum (ER) membrane and it is regulated in response to phosphorylation by casein kinase II (CK2), an enzyme that promotes cell division [11]. ZIP7 is a gatekeeper of cytosolic zinc release from subcellular organelles including the ER and Golgi apparatus [12]. It has been demonstrated that genetic ablation of *ZIP7* resulted in reduced cytosolic zinc levels, and abnormalities in cell proliferation and ER function in human osteosarcoma cell lines [13]. Similarly, dysfunctional ZIP7 caused proliferation of the tamoxifen-resistant MCF-7 breast cancer phenotype [14]. Recent data on zinc transporters also suggests that Zip7 is implicated in glucose metabolism and glycemic control in skeletal muscle cells [15]. The ablation of *Zip7* in skeletal muscle cells resulted in a substantial reduction in several genes and proteins involved in glucose homeostasis. These included the phosphorylation of Akt, the insulin receptor (Ir), insulin receptor substrates 1 and 2 (Irs1 and Irs2), the glucose transporter Glut4, and glycogen branching enzyme (Gbe). Similarly, studies identified a redistribution of cellular ER zinc in hyperglycemic rat heart cells that involved changes in Zip7 protein and Zip7 phosphorylation [16]. Given the role of Zip7 in regulating zinc flux and the activation of key cell signaling molecules associated with glucose metabolism, we propose that this transporter controls cell signaling pathways involved in glucose metabolism in skeletal muscle.

## 2. Materials and Methods

### 2.1. Cell Culture

Mouse C2C12 cells were obtained from Professor Steve Rattigan, Menzies Institute for Medical Research, Hobart, Australia. C2C12 cells were cultured in Dulbecco’s Modified Eagle Medium (DMEM) (Thermo Fisher, Victoria, Australia) medium that contained 10% fetal calf serum (FCS) and 100 U/mL penicillin/streptomycin (Thermo Fisher) and were maintained at 37 °C and 5% CO_2_ in a humidified atmosphere. C2C12 cells were differentiated into myotubes by the addition of media containing 2% horse serum (Thermo Fisher) for seventy-two hours. The cells were then exposed to serum-free conditions for three hours prior to the different treatments as outlined below.

### 2.2. Protein Extraction

Whole cell protein lysates were prepared in RIPA Lysis buffer in the presence of protease and protein phosphatase inhibitors (Thermo Fisher) as previously described [17]. Briefly, whole cell lysates were vortexed every 10 min for 1 h on ice and centrifuged at 15,000 rpm for 5 min. The protein concentrations of the supernatants were determined by a BCA assay kit as per manufacturer’s instructions (Thermo Fisher).

### 2.3. RNA Extraction

Total RNA was extracted using the Qiagen RNeasy Mini Kit as per manufacturer’s instructions (Qiagen, Victoria, Australia). Briefly, cells were lysed in RLT Buffer, placed directly into a QIAshedder spin column and centrifuged for 2 min. Lysates were then passed through a RNeasy spin column and purified by adding RW1 and RPE buffer. The purified RNA was eluted in RNAse-free water and total RNA concentration was determined by UV spectrometry.

### 2.4. cDNA Synthesis

Complementary DNA (cDNA) was synthesized from extracted total RNA using a High-Capacity cDNA Reverse Transcription Kit (Thermo Fisher, Victoria, Australia) and using random hexamers according to the manufacturer’s instructions. Briefly, 10 µL cDNA reverse-transcription mix was added to 10 µL genomic DNA elimination mix and incubated at 42 °C for 15 min. The reaction was stopped by incubating the samples at 95 °C for 5 min. The resulting reverse transcription products were stored at −20 °C until use.

### 2.5. Mice and Diets

The experimental procedures for all animal work has been previously described and the mice used in these experiments represent a sub group of a previously published cohort of animals [18]. All experiments involving the use of animals for research were approved by the Alfred Medical Research Education Precinct Animal Ethics Committee and were conducted in accordance with the National Health and Medical Research Council of Australia guidelines. Ethics number E/1255/2012/B. Mice (six mice per group) were fed either a normal chow (NC) diet (14.3 MJ/kg, 76% of kJ from carbohydrate, 5% fat, 19% protein) or a high-fat diet (HFD), (19 MJ/kg, 36% of kJ from carbohydrate, 43% fat [42.7% saturated, 35.1% monounsaturated and 21.7% polyunsaturated fatty acids] and, 21% protein), Specialty Feeds, Glen Forrest, Western Australia, Australia). During the experiment, the animals were given their prescribed diet and water ad libitum, except for fasting periods before a glucose tolerance test and were housed at 22 °C on a 12-h light/dark cycle.

### 2.6. Body Composition Analysis

Body mass was measured using standard laboratory scales (Mettler Toledo, Greifensee, Switzerland). Lean and fat mass was measured using a 4-in-1 EchoMRI (Houston, TX, USA). 

### 2.7. Oral Glucose Tolerance Test

Mice were fasted for 6 h and subsequently received an oral gavage of 2 g glucose/kg lean body mass (25% *w*/*v* glucose solution). An oral glucose tolerance test (OGTT) was performed and glucose levels were measured from tail tip blood at 0, 15, 30, 45, 60, 90 and 120 mins.

### 2.8. Mouse Tissue Collection and Protein Extraction

Mice were sacrificed by sodium pentobarbital overdose. Skeletal muscle quadriceps were used for analysis. Skeletal muscles used for protein analysis were frozen on dry ice and stored at −80 °C until analysis. A protease inhibitor cocktail was added to the CelLytic MT (Mammalian Tissue Lysis/Extraction Reagent, Sigma) reagent and a ratio of tissue to CelLytic MT reagent of 1:20 was used for tissue protein extraction. Samples were then transferred to a pre-chilled microhomogenizer and tissues were homogenized. The lysed samples were centrifuged for 10 min at 12,000–20,000× *g* to pellet the tissue debris. The protein- containing supernatants were transferred to a chilled test tube and stored at −80 °C. Protein concentrations of the supernatants were determined using the BCA assay kit as per manufacturer’s instructions (Thermo Fisher).

### 2.9. Fatty Acid Preparation and Cell Culture Treatment

Palmitate was prepared as previously described [19]. Briefly, fatty acid was mixed with ethanol to a final concentration of 100 mM. Then the mixture was sonicated on ice at 200W with 10-sec bursts, 3-sec off pulses until the mixture became a milky homogenous solution. Prepared fatty acid stock was kept at 4 °C and protected from light. For fatty acid treatment, the fatty acid stock solution was added to the cell culture medium containing 2% horse serum at 60 °C to a final concentration as stated. For the control, the same amount of ethanol was added to the cell culture medium containing 2% horse serum. Cells were cultured with increasing concentrations of palmitate-containing media for 24 h followed by 10 nM insulin treatment for 30 min prior to cell lysate harvest. Following treatment, whole cell lysates were prepared and immunoblot analysis was performed on pAkt, Zip7, glucose transporter 4 (Glut4), phospho-tyrosine and Gapdh.

### 2.10. Cell Viability Assay [1-(4,5-Dimethylthiazol-2-yl)-3,5-diphenylformazan] Assay

Cell viability was measured using the MTT (3-(4,5)-dimethylthiahiazo(−z-y1)-3,5-diphenytetrazoliumromid) assay as per manufacturer’s instructions (Thermo Fisher). Briefly, C2C12 skeletal muscle cells were seeded at a density of 2 × 10^4^ cells/well in a 96-well plate. Following C2C12 differentiation the cells were treated with varying concentrations of palmitate (as described in the results) for 24 h. Then 15 μL of 5 mg/mL MTT was added to each well. Following a 4 h incubation at 37 °C, the produced formazan was solubilized in 150 μL dimethyl sulfoxide (DMSO). The absorbance was measured at 490 nm using a microplate reader (TECAN infinite M200 PRO, Männedorf, Switzerland).

### 2.11. Insulin Receptor Inhibition

Insulin receptor tyrosine kinase activity is inhibited by 50 μM insulin receptor tyrosine kinase inhibitor hydroxy-2-naphthalenylmethylphosphonic acid trisacetoxymethyl ester (HNMPA-(AM)3) after 1 h. It suppresses the activation of pAkt after 30 min of insulin treatment [17]. C2C12 skeletal muscle cells were treated with the insulin receptor inhibitor [HNMPA-(AM)3] (Abcam, Victoria, Australia) for 1 h at 0, 25 and 50 μM. This was followed by treating the C2C12 cells with 10 nM insulin for 30 min. Following treatment, whole cell protein lysates were extracted and immunoblot analysis was performed on pAkt, Zip7, glucose transporter 4 (Glut4) and Gapdh.

### 2.12. Western Blotting

Tissue or cell-extracted proteins (20 µg) were electrophoresed using 4–15% SDS-polyacrylamide gels (Bio-Rad, New South Wales, Australia) and immunoblotted using semi-dry electrophoresis blotting system. Phosphorylated Akt (1:5000), Zip7 (1:1000) or Glut4 (1:1000) (Cell Signaling Technology, Beverly, MA, USA) primary antibodies were used for immunoblotting. Antibodies were detected with HRP-linked secondary antibodies (1:5000) (Cell Signaling Technology, USA). Then, membranes were stripped with Restore Plus Western Blot Stripping Buffer (Thermo Fisher) and reprobed for the related housekeeping gene (Gapdh 1:2000 or total Akt 1:5000, Cell Signaling Technology, USA). Proteins were visualized with SuperSignal West Femto Kit (Thermo Fisher) and imaged using an Odyssey infrared imaging system (LiCor, Millennium Science, Victoria, Australia). Relative band density was quantified using Image J software. Representative immunoblots are provided. Experiments were repeated three times and statistical analysis was performed on the signal intensity of the protein of interest relative to the house keeping gene on all protein bands from each of the experiments.

### 2.13. Zip7 Overexpression

To determine the role of Zip7 in modulating cellular pathways, a plasmid-based system was constructed that overexpressed the Zip7 protein (Origene, Rockville, MD, USA, Clone ID MR216531; plasmid cytomegalovirus, pCMV-Zip7). The vector was transformed into high-efficiency bacterial cells and plasmid DNA was isolated using an ISOLATE Plasmid Kit. The bacterial transformation was performed by heat-shock at 42 °C for 30 s as described by the manufacturer’s instructions (Thermo Fisher). The transformed bacteria were plated onto LB agar plates containing appropriate antibiotics and incubated overnight at 37 °C. Bacterial colonies were harvested and plasmids isolated using an ISOLATE II plasmid midi kit as per the manufacturer’s instructions (Bioline, New South Wales, Australia). Restriction digest analysis using the enzymes Sgf1 and and Mlu1 was used to determine the correct insertion of the *Zip7* gene from the plasmid. *Zip7* containing plasmids were transfected into skeletal muscle cells using Lipofectamine 3000 reagent (Thermo Fisher) as per manufacturer’s instructions. Briefly, skeletal muscle cells were cultured as previously described in 6-well dishes. Once the cells had reached 70% confluence, approximately 1 μg of pCMV-*Zip7* (and pCMV empty vector) was mixed with the Lipofectamine 3000 in DMEM and placed on the cells. After 24 h the cells were harvested for RNA and protein. The quality and quantity of RNA were assessed by nucleic acid spectrometry (Bio-Rad) and the measurement of optical density at 260 and 280 nm.

### 2.14. Quantitative Real-Time PCR (qPCR)

To determine whether Zip7 is overexpressed in skeletal muscle cells, qPCR was performed as previously described [15]. qPCR was performed using 20 ng of cDNA template with 2 x Sensi Fast Sybr (Bioline) as per manufacturer’s instructions. The relative level of target gene expression was normalized to eukaryotic elongation factor 2 (Eef2). Primers to amplify the Zip7 sequence were designed using PrimerFind (NCBI: https://www.ncbi.nlm.nih.gov). Sequences of the primers were as follows: Zip7 (forward: 5′- CGC ATG CCT TGG AAC CTC AT- 3′; Reverse: 5′- GGC GAC AAT CCC ACT GAG AA-3′), Eef2 (Forward: 5′- CAC AAT CAA ATC CAC CGC CAT-3′; Reverse: 5′- TGG CCT GGA GAG TCG ATG A- 3′) (Integrated DNA Technologies, Singapore Science Park II, Singapore). RT^2^ qPCR Primer Assay for Mouse Slc2a4, (Qiagen), was used to amplify the Glut4 sequence.

### 2.15. Insulin Signaling Pathway Array

Insulin Signaling Pathway Array (SABiosciences, Qiagen) was utilized to detect the expression of 84 genes implicated in the insulin signaling pathway. cDNA from the control (empty vector; pCMV) and pCMV-*Zip7* transfected C2C12 cells was assayed across the array. Real-time PCR was performed to analyze the expression of a focused panel of genes related to insulin-responsiveness and results were analyzed using GeneGlobe Data Analysis Center (Qiagen, Australia).

### 2.16. Data Analysis

Results are presented as means ± SDs. Statistical comparisons were performed using Student’s *t*-test (Prism GraphPad, version 8, San Diego, CA, USA) for mRNA expression and protein analysis. A significant effect was demonstrated at *p* < 0.05. For mouse studies, differences between normal chow (NC) and high-fat diet (HFD) (diet effect) were analyzed by a paired *t*-test. For the OGGT, two-way analysis of variance (ANOVA) with a *post-hoc* analysis (Bonferroni) was used to detect the effect of diet on glucose clearance. Analysis was performed by Prism GraphPad (version 8). A *P* value less than 0.05 was considered statistically significant.

## 3. Results

### 3.1. Glucose Regulates Zip7 Expression in C2C12 Skeletal Muscle Cells

**Figure 1:**
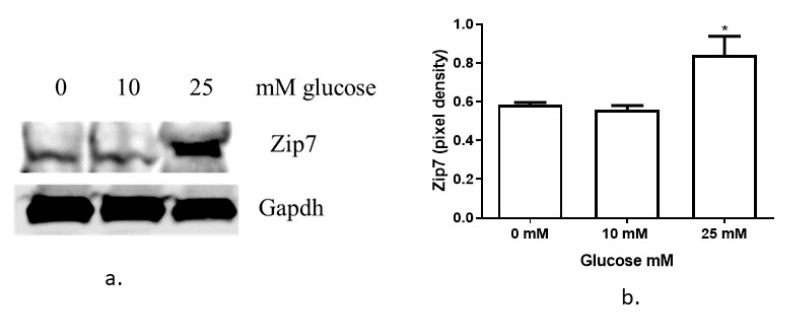
Analysis of Zip7 in mouse skeletal muscle cells treated with glucose. (**a**) Increasing concentrations of glucose (0, 10 and 25 mM) were added to C2C12 cells over 24 h. Gapdh was used as an internal loading control and levels of Zip7 were normalized to Gapdh. Representative image of three independent western blot assays that were performed on three independent treatments (*n* = 3). (**b**) Densitometry graphs for Zip7 from three independent data westerns blots. * = *p* < 0.05.

Previously we have shown that reduced *Zip7* in C2C12 skeletal muscle cells led to a significant decrease in the phosphorylation of Akt, reduced Glut4 expression and compromised insulin-mediated glycogen synthesis [15]. Accordingly, we wanted to test whether glucose could regulate Zip7 in C2C12 skeletal muscle cells to establish a role for this transporter in glycemic control. Previous studies have reported that Zip7 is upregulated by glucose in rat cardiomyocytes [16] and mouse pancreatic islets [20]. Accordingly, treatment of C2C12 cells with 0, 10 and 25 mM glucose for 2 h resulted in a robust increase in the protein expression of Zip7 (Figure 1a,b).

### 3.2. The Expression of Zip7 and Glut4 Is Suppressed in Insulin-Resistant C2C12 Cells Treated with Either an Insulin Receptor Inhibitor Hnmpa-(Am)3 or Palmitate

**Figure 2:**
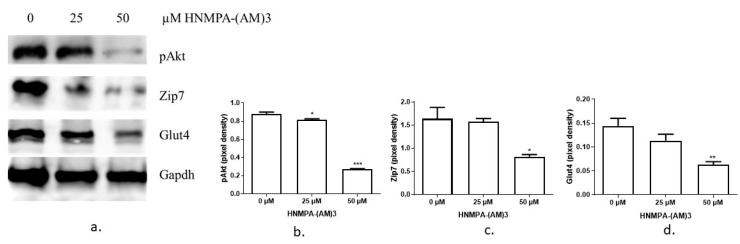
Western blot results of pAkt, Zip7 and Glut4 expression in C2C12 cells treated with insulin receptor tyrosine kinase inhibitor HNMPA-(AM)3. (**a**) The inhibitor concentration was 0, 25 and 50 µM. From the top the proteins are, pAkt, Zip7, Glut4 and Gapdh. Gapdh was used as an internal loading control. Three independent western blots on three independent treatments were performed (n = 3). (**b**–**d**). Densitometry graphs for pAkt, Zip7 and Glut4, respectively from three independent data westerns blots. * = *p* < 0.05, ** = *p* < 0.01, *** = *p* < 0.001.

Given that Zip7 could be regulated by glucose in C2C12 skeletal muscle cells, we decided to perform several studies to recapitulate a compromised glucose state in skeletal muscle by creating insulin-resistant C2C12 cells. To recapitulate an insulin-resistant state in skeletal muscle we utilized two experimental procedures: (1) an inhibitor of the insulin receptor and, (2) palmitate treatment [19,21,22]. Initially we tested the ability of the insulin receptor inhibitor HNMPA-(AM)3 to inhibit the insulin signaling pathway by measuring the downstream target of the insulin receptor, Akt. It has been previously shown that 50 µM of HNMPA-(AM)3 is sufficient to inhibit insulin-induced pAkt in C2C12 cells [19]. Accordingly, 50 µM of HNMPA-(AM)3 was sufficient to inhibit the insulin-dependent activation of pAkt in C2C12 skeletal muscle cells (Figure 2a,b). We also assayed the levels of Zip7 and Glut4 in the HNMPA-(AM)3 treated C2C12 cells and identified a significant reduction in the levels of these proteins (Figure 2a,c,d).

**Figure 3:**
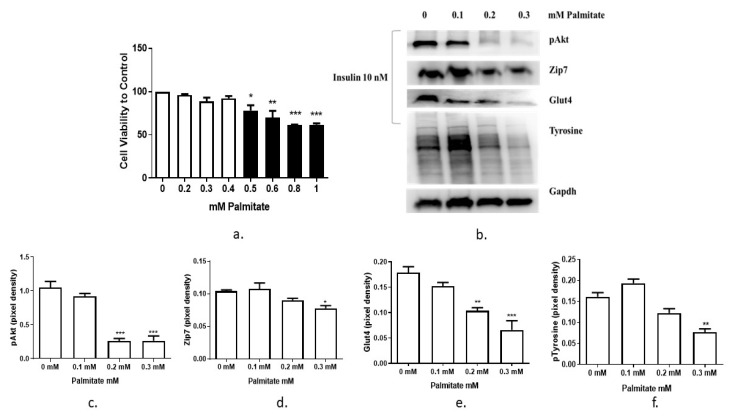
Recapitulation of an insulin-resistant state with palmitate. (**a**) MTT assay for cell viability assay in C2C12 cells treated with increasing concentrations of palmitate. Concentrations of palmitate are 0, 0.2, 0.3 and 0.4 mM (white bars); 0.5, 0.6. 0.8 and 1 mM (black bars). Cell viability was performed three times (*n* = 3). (**b**) Palmitate-induced insulin resistance in C2C12 cells. Cells were treated with 0, 0.1, 0.2 and 0.3 mM palmitate over 24 h, then stimulated with 10 nM insulin for 30 min before harvesting total protein. Phosphorylated Akt (Ser473), Zip7, Glut4 and phosphor-tyrosine were immunoprobed by western blotting. Gapdh was used as an internal control. Three independent western blots on three independent treatments were performed (*n* = 3). (**c**–**f**). Densitometry graphs for pAkt, Zip7, Glut4, and pTyrosine, respectively from three independent data westerns blots * = *p* < 0.05, ** = *p* < 0.01, *** = *p* < 0.001.

It has been shown previously that saturated fatty acid palmitate reduces insulin sensitivity and induces insulin resistance in skeletal muscle [18]. In this experiment, the inhibitory effect of palmitate on the insulin signaling pathway in C2C12 myotubes was tested. Initially we aimed to test the viability of the C2C12 cells following palmitate treatment. The result of the MTT assay showed that 0.3 mM of palmitate did not significantly suppress C2C12 cell viability (Figure 3a), while at the same time, it suppressed insulin-stimulated phosphorylation of Akt (Figure 3b,c). Accordingly, we chose 0.3 mM of palmate as the highest concentration of this fatty acid in the following experiments.

To determine the effect of palmitate treatment on Zip7 we assayed the level of Zip7 protein in palmitate-treated C2C12 cells. The expression level of Zip7 protein were reduced when treated with increasing concentrations of palmitate (Figure 3b,d). We also observed a reduction in pAkt in the presence of palmitate as previously described [19]. Glut4 was also reduced in the presence of increasing palmitate concentrations (Figure 3b,e). We also tested the ability of palmitate to reduce the levels of phospho-tyrosine, a key amino acid involved in cellular signal transduction. Following palmitate treatment, the levels of phospho-tyrosine were also reduced (Figure 3b,f). These results indicate that palmitate treatment was sufficient to induce an insulin-resistant state in C2C12 skeletal muscle cells (as evidence by the reduction in pAkt and phospho-tyrosine).

### 3.3. Testing the Function of ZIP7 in Controlling Genes Involved in Insulin Signaling 

**Figure 4:**
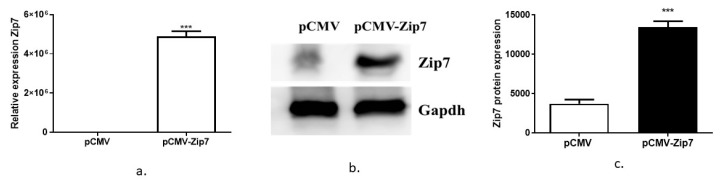
Overexpression of Zip7 in C2C12 skeletal muscle cells. (**a**) Quantitative real-time polymerase chain reaction (PCR) for the overexpression of Zip7 mRNA in pCMV versus pCMV-Zip7. (**b**) Western blot for pCMV-Zip7 protein. Gapdh was used as a loading control. (**c**) Densitometry results from the western blot data pCMV versus pCMV-Zip7. The experiments were performed three times and statistical significance compared ZIP-7 over-expressed group to the control. *** = *p* < 0.001.

The above studies suggest that Zip7 is involved in metabolic processes that could enhance cell signaling pathways associated with glucose metabolism. Accordingly, we wanted to test whether Zip7 could activate genes implicated in insulin signaling and glucose homeostasis. Initially we overexpressed Zip7 in C2C12 skeletal muscle cells utilizing an overexpression plasmid pCMV (Figure 4). We observed a significant increase in pCMV-*Zip7* mRNA compared to pCMV control (Figure 4a). Similarly, we observed a significant increase in pCMV-Zip7 protein compared to pCMV control (Figure 4b,c).

To further identify the effect of pCMV-Zip7 overexpression on insulin signaling, we used an insulin signaling pathway array, which has the targeted expression of eighty-four genes involved in insulin signaling and glucose metabolism. The genes were selected based on a commercially available insulin signaling array (SABiosciences, Qiagen) and focused cellular pathways. We tested the pCMV-Zip7 overexpression plasmid to target the expression of genes on this array. Following transfection of the pCMV-Zip7 overexpression plasmid and control empty vector (pCMV) in C2C12 cells, total RNA was extracted, and cDNA synthesis performed. Changes in the expression of all genes on the array following pCMV-Zip7 overexpression transfection are presented as (1) the gene name, (2) fold up or down regulation, and (3) the *p*-value (Table 1). Although we identified several genes with fold-change expression greater than the statistically significant highlighted genes in Table 1 below (in bold), these did not reach significance and did not meet the stringent data normalization processes (GeneGlobe Data Analysis Center Qiagen).

**Figure 5:**
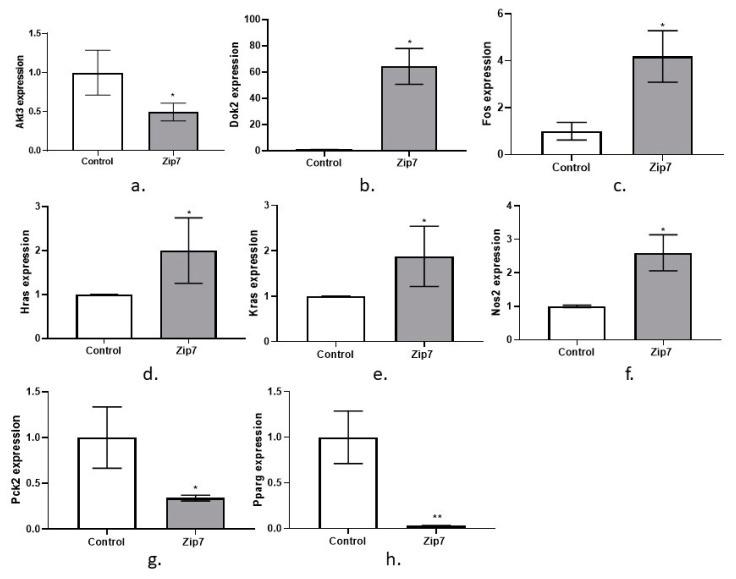
Quantitative real time PCR on an insulin signaling RT-Profiler Array for pCMV-Zip7 overexpression. Genes are (**a**) Akt3, (**b**) Dok2, (**c**) Fos, (**d**) Hras, (**e**) Kras, (**f**) Nos2, (**g**) Pck2, (**h**) Pparg. Control = pCMV empty vector. Zip7 = overexpression pCMV-Zip7. * *p* < 0.05, ** *p* < 0.01.

Representative graphs are given for the genes that were significantly changed due to the over expression of pCMV-Zip7. These include Akt3, Dok2, Fos, Hras, Kras, Nos2, Pck2, and Pparg, (Figure 5a–h).

### 3.4. The Expression of Zip7 and Glut4 Is Reduced in the Skeletal Muscle of Mice Fed a High-Fat Diet

**Figure 6:**
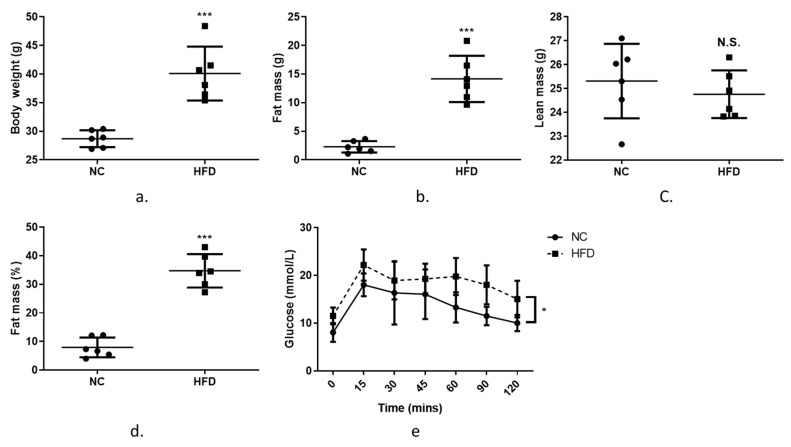
Characteristics of mice fed a normal chow (NC) diet or a high-fat diet (HFD) for 10 weeks. (**a**) body weight (g), (**b**) fat mass (g), (**c**) lean mass (g), (**e**) fat mass (%), (f) plasma glucose levels over 120 min following an oral gavage of 2 g glucose/kg lean body mass (25% *w/v* glucose solution). Unpaired *t*-test was used for the NC versus the HFD comparisons (**a**–**d**) and a two-way repeated measure ANOVA was used for the OGTT (**e**). Graphs indicate mean ± SD. * = *p* < 0.05. *** *p* < 000.1.

The results above suggest that Zip7 has a role in modulating genes involved in glucose metabolism and is reduced in an insulin-resistant state when treated pharmacologically or with fatty acids. This suggests that Zip7 could be regulated by changes in fatty acids or a high-fat diet, for example. To determine if Zip7 is modulated by a high-fat diet we performed experiments on C57BL/6J mice consuming a normal chow (NC) or high-fat diet (HFD) for 10 weeks. As expected on the HFD, body weight, fat mass and body fat percentages were increased compared to the NC-fed mice while lean mass remained unchanged (Figure 6a–d). Next, we performed an oral glucose tolerance test (OGTT) to assess insulin-stimulated glucose clearance. While blood glucose decreased over a 120-min time course in both NC and HFD cohorts, there was an overall dietary effect on glucose clearance in the HFD mice versus the NC control (Figure 6e).

**Figure 7:**
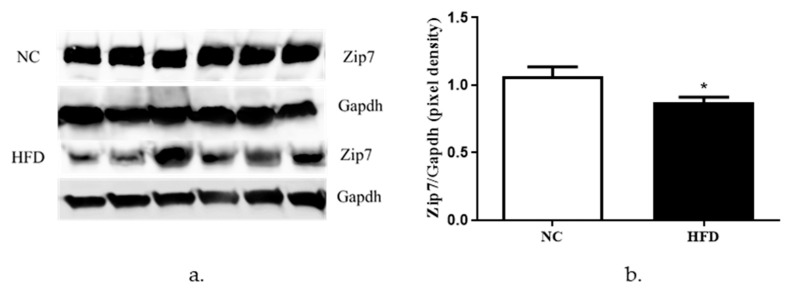
Analysis of Zip7 in mouse skeletal muscle tissues in NC versus HFD mice. Gapdh was used as an internal loading control and levels of Zip7 were normalized to Gapdh. (**a**) Western blot for Zip7 in NC versus HFD. (**b**) Densitometry of the western blot results. The experiments were performed on six animals per group (*n* = 6) and statistical significance was compared between the HFD to the NC mice group. * = *p* < 0.05.

To determine the protein expression of Zip7 in NC versus HFD-fed mice, we performed western blots on proteins extracted from quadricep skeletal muscle tissue from both cohorts. We observed a significant decrease in the expression of Zip7 and Glut4 in the HFD mice groups relative to control mice (Figure 7a,b).

**Figure 8:**
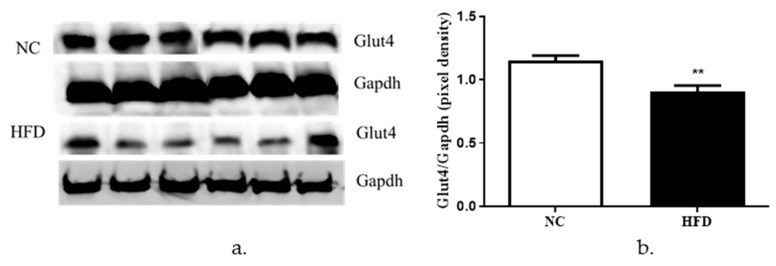
Analysis of Glut4 in NC versus HFD mouse skeletal muscle tissues. Gapdh was used as an internal loading control and levels of Glut4 were normalized to Gapdh. (**a**) Western blot for Glut4 in NC mice versus HFD. (**b**) Densitometry of the western blot results. The experiments were performed on six animals per group (*n* = 6) and statistical significance was compared between the HFD to the NC mice group. ** = *p* < 0.01.

We also measured the protein levels of Glut4 in the NC versus the HFD mice. We observed a significant reduction in the protein levels of Glut4 in the HFD mice when compared to the NC mice (Figure 8a,b).

## 4. Discussion

We have previously demonstrated the effect of zinc as an essential trace element implicated in the insulin signaling pathway [17]. However, its mechanism of action in the insulin signaling pathway and glucose uptake has not been determined. Insulin resistance, a hallmark of T2D [23], is characterized by compromised insulin-mediated activation of the PI3K/Akt pathway regulating glucose uptake via Glut4 transporters [24]. Skeletal muscle is the major site of peripheral insulin resistance of which much of the complexity of this disorder remains undefined, including the role of zinc and zinc transporter Zip7 in insulin signaling and glucose metabolism. Previous investigations showed that zinc has insulin mimetic activity leading to increased total glucose consumption [17]. Glucose transporters are important proteins that regulate glucose uptake through cellular membranes. In this regard, Glut4 facilitates the transport of glucose in skeletal muscle [25]. Glut4 translocation to the cell membrane from specialized storage vesicles in the cytosol by insulin is a rate-limiting step of glucose disposal [26]. Accordingly, treatment of L6 rat skeletal muscle cells with zinc induced Glut4 translocation in a dose-dependent manner [26]. These studies showed that zinc exerted insulin-like effects by phosphorylation of Akt and subsequent mobilization of glut4.

Several studies have shown that disturbances in zinc transporter function and consequently zinc signaling lead to cellular and physiological disturbances. For example, zinc transporter ZnT8-deficient mice exposed to a high-fat diet develop severe insulin resistance and obesity [27]. Similarly, Zip13-deficient mice have enhanced beige adipocyte production and energy expenditure and this was concomitant with resistance to HFD-induced obesity, and improved glucose and insulin tolerance [28]. Other studies have shown that ZIP7 (and ZIP6) play an important role in regulating zinc homeostasis in MIN6 pancreatic beta cells [29]. These studies identified a compensatory increase in the expression of ZIP7 in the presence of a targeted ZIP6 knockdown. It was suggested that ZIP6 and ZIP7 transporters orchestrate cytosolic zinc flux by increasing extracellular zinc uptake or by releasing ER-stored zinc into the cytosol when required. This is supported by studies where ZIP7 knockdown in human osteosarcoma (MG-63) cells exhibited an increase in ER zinc and a decrease in cytosolic zinc [13]. Moreover, other studies have identified a dual role for ZIP7 and ZnT7 in cardiomyocytes [16]. Here it was identified that changes in the expression of ZIP7 and ZnT7 induces ER stress through the loss of ER zinc during hyperglycemic conditions. These authors also suggest that perturbations in the expression of ZIP7 and ZnT7 may lead to decompartmentalization of zinc across the ER and therefore lead to persistent ER stress.

In the context of Zip7, ablation of this transporter in mouse skeletal muscle cells resulted in reduced Glut4 protein and a reduction in insulin-stimulated glycogen synthesis [15]. To expand on this study, we examined the protein levels of both Zip7 and Glut4 in an *in vitro* insulin-resistant skeletal muscle cell model and an *in vivo* skeletal muscle of mice fed normal chow (NC) versus a high-fat diet (HFD).

First, we treated C2C12 skeletal muscle mouse cells with glucose to recapitulate hyperglycemic conditions and measured the protein levels of Zip7. We observed a significant increase in the protein levels of Zip7 in cells treated with increasing concentrations of glucose. This result is supported by studies showing high glucose induces the expression of Zip7 mRNA in mouse pancreatic islets [20] and Zip7 mRNA and protein in rat cardiomyocytes [16].

Second, we aimed to recapitulate an in vitro insulin-resistance cell culture model and investigate the protein levels of Glut4 and Zip7. C2C12 skeletal muscle cells were treated with HNMPA-(AM)3, an inhibitor of insulin receptor tyrosine kinase activity, and palmitate. HNMPA-(AM)3 abolished the expression of insulin-induced pAkt and is supported by previous studies [17,30,31]. We observed a clear reduction in Zip7 and Glut4 protein upon treatment with HNMPA-(AM)3. To our knowledge no data exists showing insulin receptor inhibition action on Zip7 expression. Similarly, palmitate-induced insulin resistance in C2C12 skeletal muscle cells was comparable with the insulin receptor inhibition study. Excessive plasma free fatty acids are associated with insulin resistance in both diabetic and non-diabetic subjects [19]. Palmitate plays a critical role in the initiation and development of insulin resistance as exposure of C2C12 cells to palmitate suppressed insulin-stimulated Akt1 phosphorylation and glucose uptake [19]. It was observed that palmitate treatment reduced insulin-induced Akt phosphorylation that is consistent with an insulin resistant state [21]. Moreover, the protein expression of Zip7 and Glut4 were reduced by palmitate and by HNMPA(AM)3. We also detected a decrease in the phospho-tyrosine expression in palmitate-treated C2C12 cells. These data suggest that Zip7 is involved in the insulin signaling pathway and glucose metabolism. While it is unclear how Zip7 contributes to the control of these pathways, there is some evidence on the potential mechanisms. For example, studies in C2C12 mouse skeletal muscle cells identified that ablation of Zip7 resulted in the modulation of key genes implicated in glucose metabolism including those of glycolysis, gluconeogenesis, the citric acid cycle, and glycogen metabolism [15]. Moreover, a reduction in the mRNA of the insulin receptor and insulin receptor substrates 1 and 2, Glut4 protein, pAkt and insulin-induced glycogen synthesis was observed.

While these studies suggest that ZIP7 is associated with the insulin-signaling axis, how this association occurs in unclear. The role of ZIP7 in initiating zinc flux from the ER into the cytosol poses several questions, and are therefore limitations of this manuscript. For example, what are the cellular concentrations of zinc during treatment with palmitate or HNMPA-(AM)3 in an insulin-resistant state? To our knowledge, there are limited studies addressing the effect of fatty acids on cellular zinc status. One study identified the fatty acids oleate, elaidate, stearate and palmitate increased zinc concentrations in human macrophages, with elaidate having the greatest effect [32]. The authors suggest the importance of this based on zinc being a major regulator of macrophage activity. Accordingly, it will be important to determine cellular zinc status in insulin resistant skeletal muscle cells and determine whether zinc signaling processes are compromised. Moreover, the role of ZIP7 in these processes needs to be addressed. For example, if ZIP7 is reduced (or defective) in an insulin-resistant state, what effect does this have in zinc mimetic activity, and glucose metabolism?

To further investigate effects of Zip7 on glucose metabolism and insulin signaling, we established a Zip7 overexpression cell culture model. Previous studies identified that a reduction in Zip7 in skeletal muscle cells resulted in changes in several genes involved in glucose metabolism [15]. To extend on these studies, a plasmid-based system that overexpressed Zip7 was utilized to determine the ability of this transporter to regulate genes involved in the insulin signaling pathway. These studies identified significant changes in several genes including *Akt3*, *Dok2*, *Fos*, *Hras*, *Kras*, *Nos2*, *Pck2*, and *Pparg.*

It was observed that *Akt3*, *Pck2*, and *Pparg* were significantly downregulated in the Zip7-overexpressing cells. The roles of Akt1 and Akt2 are well established in metabolism, however there is less known about Akt3 [33]. It is suggested that Akt3 may play a role in defects in insulin signaling that occur in insulin resistant states [34].

There are two Pck isoenzymes, phosphoenolpyruvate carboxykinase (PEPCK-C, cytoplasmic) encoded by *Pck1* and PEPCK-M (mitochondrial), encoded by *Pck2* (mitochondrial). Although the role of *Pck1* has been extensively studied for its role in gluconeogenesis in the liver, *Pck2* remains somewhat unknown [35]. However, these data for *Pck2* are consistent with previous studies where a reduction in Zip7 mRNA led to an increase in this gene in C2C12 skeletal muscle cells [15].

*Pparg* (peroxisome proliferator activated receptor gamma) is a member of the nuclear hormone superfamily of transcription factors that modulates genes involved in insulin signaling and lipid metabolism [36]. While *Pparg* expression is low in skeletal muscle, C2C12 skeletal muscle cells stably overexpressing Pparg resulted in increased glucose uptake and inhibition of *Pparg* expression induced insulin resistance as determined by reduced deoxyglucose uptake [36]. In contrast, mice heterozygous for *Pparg^+/-^* displayed greater insulin sensitivity than the wild-type animals [37] and improved insulin resistance and obesity [38]. While these studies counter the current dogma on Pparg action in metabolism, it is not clear why *Pparg* expression is reduced in our overexpression Zip7 studies. A recent study identified Zip7 was upregulated by ethanol exposure in the livers of mice and this was concomitant with a downregulation of another *Ppar* family member, *Ppara* [39]. In C2C12 skeletal muscle cells with an ablation of *Pparg*, it was identified that myogenic differentiation was compromised [40]. These studies suggest that *Pparg* is critical for the differentiation of skeletal muscle myoblast to myotubes and perhaps Zip7 overexpression overrides the mechanism of Pparg-inhibitory effect on the myogenic phenotype. Support for a Zip7-mediated role in promoting myogenic differentiation was recently identified [41]. It was identified that zinc promotes myoblast proliferation and differentiation of mature myotubes via Zip7 activation and the modulation of the Pi3k/Akt pathway in C2C12 skeletal muscle cells.

We also observed significant upregulation of several genes in the Zip7 overexpression cell model including *Dok2*, *Fos*, *Hras*, *Kras*, and *Nos2.* Dok2 (docking protein 2), along with its closest homologue Dok1 work as adapter proteins that recruit and assemble multiple SH2-containing molecules including p120 rasGAP and Nck [42]. There is little information on the role of Dok2 in skeletal muscle or insulin resistance, however experiments in mice lacking Dok1 or Dok2 showed enhanced expression of Erk and Akt in myeloid cells [43]. Similarly, Dok2 significantly inhibited insulin-stimulated Akt phosphorylation in COS7 cells and these authors suggested that Dok2 acts as an inhibitor of insulin action [44]. These data therefore suggest that a reduction in Dok2 would have an enhancing effect on insulin signaling.

c-Fos (FBJ osteosarcoma oncogene) is a protooncogene that dimerizes with c-Jun to form the activator protein AP1 transcription complex [45]. C-Fos is implicated in several molecular mechanisms that contribute to cellular processes including proliferation, differentiation, and apoptosis [46]. Recently, parathyroid hormone-related peptide (PTHrP) induced *c-Fos* expression was significantly reduced in Zip14-KO chondrocytes [47]. In chondrocyte differentiation, PTHrP induces phosphorylation of cAMP and the subsequent regulation of *c-Fos* via phosphorylation of the cAMP response element-binding protein (CREB) [47]. These studies suggest that Zip14 controls basal cAMP levels and its role may provide a mechanism for the zinc-mediated regulation of endocrine signaling. Whether Zip7 controls similar cAMP-mediated events (or whether Zip7 is regulated by cAMP) in skeletal muscle is yet to be determined. Zinc itself affects a wide range of second messenger and signaling molecules including Ca^2+^/calmodulin-dependent protein kinase II [48], Erk1/2, protein tyrosine phosphatase [17] and cAMP-dependent protein kinase [49]. Future studies eliminating Zip7 in skeletal muscle for example could provide valuable insight into the specific pathways controlled by Zip7-mediated zinc flux.

We also identified an increase in the expression of *Hras* (Harvey rat sarcoma virus oncogene) and *Kras* (Kirsten rat sarcoma viral oncogene homolog) in the Zip7 overexpressing skeletal muscle cell lines. *Hras* and *Kras* are canonical *ras* genes and are critical components of cell signaling pathways that control proliferation, survival, and differentiation [50]. Ras activity induces Raf and subsequent regulation of protein kinases Mek1/2 which phosphorylate ERK1/2. Mostly, the Ras/Raf/Mek/ERK pathway is critical for cell proliferation and differentiation [51].

Finally, we assayed the expression levels of Zip7 in an *in vivo* HFD mouse model. Our results revealed a decrease in the protein expression of both Zip7 and Glut4 in the skeletal muscle of HFD-fed mice compared to the NC controls. While there are no other studies reporting this observation for reduced Zip7 in skeletal muscle of HFD-fed mice, studies on obesity-associated inflammation in mammary gland tissue found HFD mice had decreased levels of Zip7. These authors suggest that inflammation in mammary tissue compromises lactation by mediating zinc retention in the endoplasmic reticulum [52]. However, several studies have assessed zinc in the context of HFD. For example, male rats fed a HFD had reduced weight gain, abdominal fat pads, plasma insulin levels, leptin and triglycerides when supplemented with zinc [53]. Similarly, it was identified that zinc supplementation improved glucose tolerance, HOMA-β, and glucose-stimulated insulin secretion in HFD mice by enhancing pancreatic β-cell function [54]. In the context of plasma zinc levels, it has been identified in obese humans and mice that leptin levels are increased with concomitant reduced plasma zinc concentrations [55]. It was suggested by these authors that reduced zinc compromises several cell signaling pathways that regulate inflammation and leptin. This is significant given that zinc-deficiency is common in obese individuals [56].

While, the connection between Zip7 and Glut4 regulation is not known, our previous data showing a reduction in Zip7 in C2C12 skeletal muscle cells led to a significant decrease in Glut4 protein and compromised insulin-mediated glycogen synthesis [15] suggests that Zip7 might be involved in skeletal muscle glycemic control. However, the mechanisms involved in these processes requires further investigation.

The studies presented suggest that Zip7 has a role in modulating insulin-signaling molecules and is reduced in an insulin-resistant state and in mice fed a HFD. While there were several changes in genes associated with Zip7 overexpression, it is unknown how these changes contribute to insulin signaling and cell signaling processes and affect glycemic control in skeletal muscle. Further studies are required to delineate the molecular mechanisms of Zip7 action on cell signaling in both insulin-resistant and diabetic in vitro and in vivo models.

## Figures and Tables

**Table 1:** Insulin signaling gene array.

Gene Name	Fold–Up or Downregulation	*p*–value
**Insulin Receptor–Associated Proteins**		
Eukaryotic translation initiation factor 4E binding protein 1 (Eif4ebp1)	−5.42	0.109812
Insulin−like growth factor 2 (Igf2)	−2.51	0.119985
Growth factor receptor bound protein 2 (Grb2)	**−1.81**	**0.044301**
Fibroblast growth factor receptor substrate 3 (Frs3)	−1.78	0.342511
Sorbin and SH3 domain containing 1 (Sorbs1)	−1.57	0.261979
Protein tyrosine phosphatase, receptor type, F (Ptprf)	−1.49	0.376424
Growth factor receptor bound protein 2−associated protein 1 (Gab1)	−1.48	0.237770
Non−catalytic region of tyrosine kinase adaptor protein 1 (Nck1)	−1.24	0.479138
Fibroblast growth factor receptor substrate 2 (Frs2)	−1.21	0.514579
Protein tyrosine phosphatase, non−receptor type 1 (Ptpn1)	−1.19	0.481621
Heat shock protein 90 alpha (cytosolic), class B member 1 (Hsp90ab1)	1.04	0.965684
src homology 2 domain−containing transforming protein C1 (Shc1)	1.13	0.659820
Protein phosphatase 1 catalytic subunit alpha (Ppp1ca)	1.15	0.595936
Casitas B−lineage lymphoma (Cb1)	1.15	0.759472
Insulin receptor substrate 1 (Irs1)	1.47	0.435010
Insulin−like growth factor I receptor (Igfr)	1.54	0.241275
Insulin receptor substrate 2 (Irs2)	1.67	0.240410
Prolactin (Prl)	1.82	0.518438
Insulin 1 (Ins1)	2.04	0.401028
Insulin−like growth factor binding protein 1 (Igfbp1)	2.14	0.391212
Neuropeptide Y (Npy)	2.60	0.057738
Docking protein 1 (Dok1)	2.91	0.103312
Docking protein 3 (Dok3)	3.30	0.315765
Growth factor receptor bound protein 10 (Grb10)	3.75	0.140065
Insulin−like 3 (InsI3)	4.33	0.287920
Thyroglobulin (Tg)	5.53	0.179122
Docking protein 2 (Dok2)	**21.79**	**0.031048**
**PI3 Kinase Signaling**		
Thymoma viral proto−oncogene 3 (Akt3)	**−2.16**	**0.019032**
Phosphoinositide−3−kinase regulatory subunit 1 (Pik3r1)	−1.57	0.350159
Thymoma viral proto−oncogene 2 (Akt2)	−1.46	0.384953
Protein kinase C, gamma (Prkcg)	−1.42	0.281971
Eukaryotic translation initiation factor 2B, subunit 1 (alpha) (Eif2b1)	−1.39	0.219592
Phosphatidylinositol−4,5−bisphosphate 3−kinase catalytic subunit alpha (Pik3ca)	−1.38	0.333187
Phosphatidylinositol−4,5−bisphosphate 3−kinase catalytic subunit beta (Pik3cb)	−1.30	0.444354
3−phosphoinositide dependent protein kinase 1 (Pdpk1)	−1.15	0.751995
Protein kinase C, iota (Prkci)	−1.14	0.707935
Mechanistic target of rapamycin kinase (Mtor)	1.08	0.874784
BCL2−like 1 (Bcl2l1)	1.21	0.631682
Phosphoinositide−3−kinase regulatory subunit 2 (Pik3r2)	1.22	0.653162
Adrenergic receptor, alpha 1d (Adra1d)	1.25	0.391856
Thymoma viral proto−oncogene 1 (Akt1)	1.36	0.241863
Dual specificity phosphatase 14 (Dusp14)	1.42	0.465154
Protein kinase C, zeta (Prkcz)	2.00	0.327070
Serine peptidase inhibitor, clade E, member 1 (Serpine 1)	2.09	0.131939
**MAP Kinase Signaling**		
Related RAS viral (r−ras) oncogene 2 (Rras2)	−1.86	0.075688
Braf transforming gene (Braf)	−1.78	0.127855
Related RAS viral (r−ras) oncogene (Rras)	−1.63	0.070002
Araf proto−oncogene, serine/threonine kinase (Araf)	−1.40	0.331800
SOS Ras/Rac guanine nucleotide exchange factor 1 (Sos1)	−1.21	0.477393
Excision repair cross−complementing rodent repair deficiency, complementation group 1 (Ercc1)	−1.02	0.884782
v−raf−leukemia viral oncogene 1 (raf1)	1.03	0.834133
Uncoupling protein 1 (mitochondrial, proton carrier) (Ucp1)	1.26	0.433977
Mitogen−activated protein kinase kinase 1 (Map2k1)	1.43	0.244012
Mitogen−activated protein kinase 1 (Mapk1)	1.82	0.134200
Kruppel−like factor 10 (Klf10)	2.12	0.143675
Nitric oxide synthase 2, inducible (Nos2)	**2.21**	**0.030364**
Harvey rat sarcoma virus oncogene (Hras)	**2.30**	**0.027193**
Ribosomal protein S6 kinase polypeptide 1 (Rps6ka1)	2.93	0.286011
FBJ osteosarcoma oncogene (Fos)	**4.42**	**0.014490**
**Carbohydrate Metabolism**		
Phosphoenolpyruvate carboxykinase 2 (mitochondrial) (Pck2)	**−2.73**	**0.046319**
Glucuronidase, beta (Gusb)	**−2.44**	**0.057494**
Pyruvate kinase liver (Pkl)	−2.00	0.361565
AE binding protein 1 (Aebp1)	−1.36	0.346200
acyl−Coenzyme A oxidase 1, palmitoyl (Acox1)	−1.23	0.517860
Hexokinase 2 (Hk2)	1.06	0.892955
Glycogen synthase kinase 3 beta (Gsk3b)	1.04	0.908617
Glyceraldehyde−3−phosphate dehydrogenase (Gapdh)	1.15	0.642638
Glucose−6−phosphatase, catalytic (G6pc)	1.22	0.432012
Glycerol−3−phosphate dehydrogenase 1 (Gpd1)	1.43	0.434684
Solute carrier family 2 (facilitated glucose transporter), member 1 (Slc2a1)	1.50	0.246541
Glucokinase (Gck)	1.96	0.316235
Fructose bisphosphatase 1 (Fbp1)	3.40	0.315244
Glucose−6−phosphatase, catalytic, 2 (G6pc2)	4.71	0.189288
**Lipid Metabolism**		
Complement factor D (adipsin) (Cfd)	−3.66	0.370569
Peroxisome proliferator activated receptor gamma (Pparg)	**−3.31**	**0.007128**
Sterol regulatory element binding transcription factor 1 (Srebf1)	**−1.59**	**0.041612**
Resistin (Retn)	−1.71	0.786857
Low density lipoprotein receptor (Ldlr)	−1.11	0.938288
Leptin (Lep)	1.31	0.490170
Solute carrier family 27 (fatty acid transporter), member 4 (Slc27a4)	1.33	0.350357
**Cell Growth and Differentiation**		
Kirsten rat sarcoma viral oncogene homolog (Kras)	**2.12**	**0.025606**
CCAAT/enhancer binding protein (C/EBP), alpha (Cebpa)	−1.41	0.700291
Vascular endothelial growth factor A (Vegfa)	−1.14	0.660055
CAP, adenylate cyclase−associated protein 1 (Cap1)	−1.13	0.673316
Jun proto−oncogene (Jun)	1.02	0.996474
Beta−2 microglobulin (B2m)	1.13	0.700177
Actin, beta (Actb)	1.28	0.577259
CCAAT/enhancer binding protein (C/EBP), beta (Cebpb)	2.33	0.145381

**Note:** Genes are listed by specific pathways: insulin receptor and associated proteins, PI3 kinase signaling, MAP kinase signaling, carbohydrate metabolism, lipid metabolism, and cell growth and differentiation. Please also note that several genes can belong to multiple pathways. The fold change for each gene within the pathway is shown initially as the highest value for the downregulation (a negative symbol) followed by a sequential increase in values into the positive range.
